# Cryopreservation and Cryobanking of Cells from 100 Coral Species

**DOI:** 10.3390/cells11172668

**Published:** 2022-08-27

**Authors:** En-Chun Toh, Kuan-Lin Liu, Sujune Tsai, Chiahsin Lin

**Affiliations:** 1 Institute of Marine Biology, National Dong Hwa University, Pingtung 944401, Taiwan; 2 Department of Post Modern Agriculture, Mingdao University, Peetow, Changhua 52345, Taiwan; 3 National Museum of Marine Biology & Aquarium, Checheng, Pingtung 944, Taiwan

**Keywords:** cryopreservation, coral, cryobank, cell, cryoprotectant, Symbiodiniaceae

## Abstract

When coral species become extinct, their genetic resources cannot be recovered. Coral cryobanks can be employed to preserve coral samples and thereby maintain the availability of the samples and increase their potential to be restocked. In this study, we developed a procedure to determine coral species-specific requirements for cryobank freezing through determining suitable cryoprotective agents (CPAs), CPA concentrations, equilibration times, holding durations, viability rates, and cell amounts for banked coral cells, and we established the first ever coral cell cryobank. Coral cells, including supporting and gland cells, epidermal nematocysts, Symbiodiniaceae and symbiotic endoderm cells (SEC) were found from the extracted protocol. Approximately half of the corals from the experimental corals consisted of spindle and cluster cells. Gastrodermal nematocysts were the least common. The overall concentration of Symbiodiniaceae in the coral cells was 8.6%. Freezing using DMSO as a CPA was suitable for approximately half of the corals, and for the other half of species, successful cell cryopreservation was achieved using MeOH and EG. EG and DMSO had similar suitabilities for *Acanthastrea*, *Euphyllia*, *Favites*, *Lobophyllia*, *Pavona*, *Seriatopora*, and *Turbinaria*, as did EG and MeOH for *Acropora*, *Echinopyllia*, and *Sinularia* and MeOH and DMSO for *Platygyra* after freezing. At least 14 straws from each species of coral were cryobanked in this study, totaling more than 1884 straws (0.5 mL) with an average concentration of 6.4 × 10^6^ per mL. The results of this study may serve as a framework for cryobanks worldwide and contribute to the long-term conservation of coral reefs.

## 1. Introduction

Coral reefs are economically, socially, and environmentally valuable. It primarily acts as a barrier that reduces waves and protects the coasts from damage. Coral reefs act as habitats, shelters, and nurseries for various marine organisms (i.e., nudibranchs, clownfish, etc.) [1,2]. In addition, coral reefs fix nitrogen and carbon by converting harmful gases to harmless gases, which act as a filtration system in the ocean [3,4]. Additionally, it may generate revenue for nations such as Australia (The Great Barrier Reef), which draws a large number of visitors each year to witness the wonderful view of marine life [2,5]. Corals can also be used for medical purposes such as anti-inflammatory, anti-cancer, bone repair, and neurological treatment [6]. However, an increasing number of factors, such as coral bleaching, coral diseases, environmental degradation, and overfishing, have led to a coral crisis [7,8]. In addition, coral bleaching has become increasingly common with climate changes due to global warming [9,10]. By 2030, 60% of the global reef area may be lost [11]. When a species of coral becomes extinct, its genetic resources cannot be recovered [12]. Coral loss can eventually lead to broad ecological effects, especially on organisms that coexist with coral and rely on it as a source of food or shelter [13].

This coral crisis has led to the emergence of coral genetic cryobanks. At cryobanks, samples are frozen and preserved at low temperatures, ensuring the year-round availability of the samples and increasing their potential to be restocked. Coral cell cryobanks have been created to preserve the genes of coral species [14]; reculture desired cells through transplantation into acceptable hosts, such as through cloning [15]; preserve the DNA and RNA of the world’s coral species [16]; study differences in coral lipid content [17]; and enable consistent access to valuable coral samples for laboratory experiments [18]. Such cryobanks provide a channel through which local organizations can expand coral nurseries and coral populations in the wild through the cryopreservation of larvae and gamete cells. The number of coral cryobanks worldwide has gradually increased, with cryobanks containing frozen samples comprising billions of *Acropora tenuis* and *Acropora millepora* sperm [19]. In addition, the Coral Hospital of the National Museum of Marine Biology and Aquarium (NMMBA) in Taiwan cryopreserved six clades of Symbiodiniaceae in 634 straws (0.25 mL) at a concentration of 1–2 × 10^6^/mL [14] and the tissues of 37 coral species in 233 vials (1.5 mL; [18,20]).

Two internationally recognized projects aim to preserve all cells, including coral cells: (1) the Frozen Ark project and (2) Global Genome Biodiversity Network. The Frozen Ark project was launched to preserve the genetic resources of threatened wild species before they become extinct. The project has been useful for conservation breeding programs. Through the project, valuable materials, such as tissues, viable somatic cells, gametes, eggs, and embryos, have been preserved. The project was made possible by international collaboration among zoos, aquariums, museums, and universities [21]. Coral tissues and Symbiodiniaceae from the NMMBA in Taiwan were indexed in the Frozen Ark. The Global Genome Biodiversity Network was established through the Memorandum of Cooperation; it is an unincorporated, international network of member organizations that share the goal of preserving high-quality, well-documented, and vouchered genomic samples of Earth’s biodiversity for research [22]. In addition, through the Genome 10K Project of 2009, the genomes of 16,203 vertebrate species were compiled by over 150 scientists to preserve the future of fish, amphibians, reptiles, birds, mammals, and ancient vertebrates [23].

The cells (e.g., somatic cells) of most species can be recultured [24], cloned through nuclear transfer [21], used to create recombinant DNA [25], introduced to host cells through gene delivery [26], or used in genome editing [27]. These methods can potentially be applied to coral cells as well. In addition to genetic engineering-related methods, other forms of technological assistance could be employed to more deeply understand coral cells, such as fluorescent protein imaging of living cells [28], coral cell cultures [29,30], ultrastructural observation [31,32], and lipid profiling [33]. Cultured cells provide key information on the DNA and RNA molecules and proteins [34].

Coral cell culture began in 1994, when Frank et al. obtained cell cultures for multiple coral species; they demonstrated that primary cells could be differentiated [35]. Coral cell culture was then expanded to other coral species (e.g., [24,29,30,36,37,38,39,40,41,42,43,44,45,46,47]. Culture media (e.g., Dulbecco’s Modified Eagle’s Medium) and antibiotic-antimycotics were applied to different coral species in well plates or petri dishes with a 12/12-h light cycle and temperature controlled at 23 °C to 26 °C. The cultures of coral species were discovered to have proliferated after 3–67 days of continuous culture. Previously, *Fungia granulosa* and *A. tenuis* reproductive or larval cells have been cultured until they reached the polyp stage [24,45]. Thus, coral cells may be cultured to save coral from extinction.

The objective of this study was to develop a procedure for determining the species-based requirements for the freezing of coral cells and establish the first coral cell cryobank. The details of the procedures for determining suitable cryoprotective agents (CPAs), CPA concentrations, equilibration time, extraction duration, viability rates, and cell number for the cryobanked coral cells are described in the following sections.

## 2. Materials and Methods

### 2.1. Coral Collection

Various wild coral species were collected from Houwan, Taiwan (N21°56.352′ E120°44.758′; N21°55.912′ E120°44.681′). The corals were transported to the NMMBA and maintained in fresh seawater in a flow-through system tank (0.6 t) with a salinity of 33–35 ppt and a flow rate of 7500 L/h, which was achieved with a wavemaker (R35210, ReefWave, Israel). The corals were broken into chunks (4 cm^2^) with chisel-like steel tools during collection. The collected wild corals were kept for a maximum duration of 7 days for the experiment. Cultured corals were obtained through husbandry at the NMMBA, and the minimum age of the cultured coral was 3 years. The coral collection was approved by the Kenting National Park Management Office.

### 2.2. Coral Identification

The corals were first identified and categorized by divers during the collection process. The categorization was confirmed at the laboratory on the basis of bone plates. The polyp and sclerite morphologies were analyzed under a light microscope (C31, Olympus, Japan), and the relevant features (e.g., corallite structure) were checked against a key to determine the species of the sample corals. The samples were maintained in a fixation buffer (10% sodium hypochlorite solution; Sigma-Aldrich, St. Louis, MO, USA) before being rinsed with distilled water and dried. The samples were subsequently transferred to a different laboratory for independent testing. The two laboratories obtained identical species-level identification results.

### 2.3. Coral Host Cell and Symbiodiniaceae Extraction

An extraction solution was prepared to separate cells from the corals. The solution comprised 3% *w/v* N-acetyl cysteine (Sigma-Aldrich) and 0.5% *w/v* trypsin (Sigma-Aldrich) in 25 mL of filtered seawater. In addition, 0.8% *w/v* NaOH (Sigma-Aldrich) was dissolved in the extraction solution to increase its pH to 8.2–8.4. The coral solutions were then shaken at 100 rpm with an orbital shaker (MS-NRK-30, Major Science, Taiwan). Color was monitored periodically to determine the extraction progress. The cells were spun (25 °C, 2000 rpm, 3 min) using a refrigerated centrifuge (5810R, Eppendorf, Germany) to wash away the extraction solution, which was replaced with filtered seawater. A 0.63 × 32 mm^2^ needle syringe (23G × 1${\frac{1}{4}}^{’’}R.B.$; Top, Japan) was used to break apart the cell chunks in the solution. The tubes (Falcon, NY, USA) were covered in aluminum foil to protect the samples from light. The coral cell types were identified based on the microscopic photography data from [48,49,50].

### 2.4. Cryopreservation

**Figure 1:**
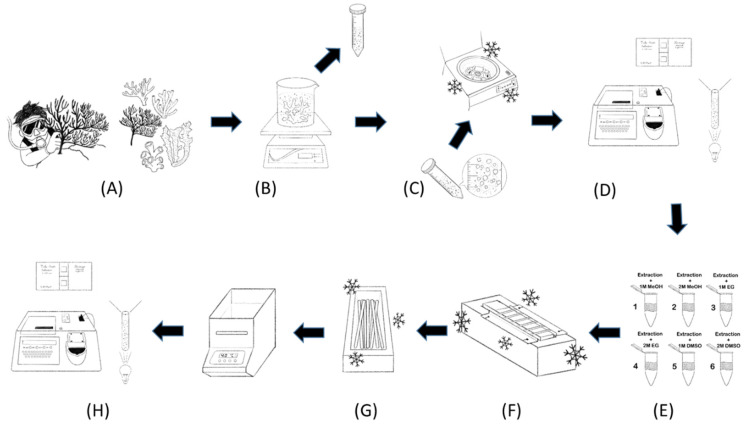
The general plan for the cryopreservation process conducted in this study. (**A**) Wild coral was collected by divers, and coral cultures were obtained through husbandry. (**B**) Coral cells were extracted. (**C**) Coral cells were centrifuged for collection. (**D**) Viability was tested using ATP assay and cell counting. (**E**) Extracted cells were mixed with different CPAs for different equilibration times. (**F**) Cells in straws were cooled through two-step cryopreservation. (**G**) Straws were soaked in liquid nitrogen and thawed in warm bath. (**H**) Cryopreservation viability test was performed.

CPAs were prepared using filtered seawater and 1 or 2 M ethylene glycol (EG; J.T. Baker, NJ, USA), methanol (MeOH; Darmstadt, Germany), or dimethyl sulfoxide (DMSO; Sigma-Aldrich). The freezing procedures entailed adding the CPAs to the samples at a ratio of 1:3. The mixtures were equilibrated at room temperature (25 °C) for 10 or 20 min, and the equilibrated samples were loaded into 0.5-mL straws (IMV Technologies, France) and suspended above liquid nitrogen for 10 min for cooling at a rate of approximately 60 °C/min on a cooling device (Taiwan patent no. M394447). The straws were immersed in a liquid nitrogen bath for at least 30 min. The straws were subsequently thawed for 10 s in a 40 °C water bath (SWB-10L-1, Major Science, Taiwan), and 1-mL samples of coral cells treated with each combination of CPA type, CPA concentration, and equilibration time were obtained for viability testing (Figure 1).

### 2.5. Viability Assay

Adenosine triphosphate (ATP) bioassays (Cellular ATP Kit HTS; BioThema, Handen, Sweden) and a hemocytometer (Neubauer-improved bright line; Marienfeld Superior, Germany) were used to test viability. An ATP viability assay can be used to determine the energy produced by cells for metabolism by using a solution that employs luciferase and D-luciferin to catalyze the release of light. For each measurement, 50 µL of sample and ATP reagent were combined in a luminometer tube and mixed for 3 min. The coral cells were then inserted into the luminometer (Lumat 9507, Berthold Technologies, Bad Wildbad, Germany) to obtain ATP readings. Cell counts were also used to assess the cell density of the samples before and after the experiments. A hemocytometer and a microscope (CX31, Olympus, Japan) were used for cell counting. The cells in a control sample were counted immediately after the extracted sample was washed, and the cells of a contrast sample were counted after the sample was thawed. A microscopy camera (518CU, ACCU-SCOPE, New York, NY, USA) and photo editing software (SE3 Micrometrics, Taiwan) were used with light microscope (CX31, Olympus, Japan) to photograph the coral cells under 400× magnification.

### 2.6. Coral Cryobanking

**Figure 2:**
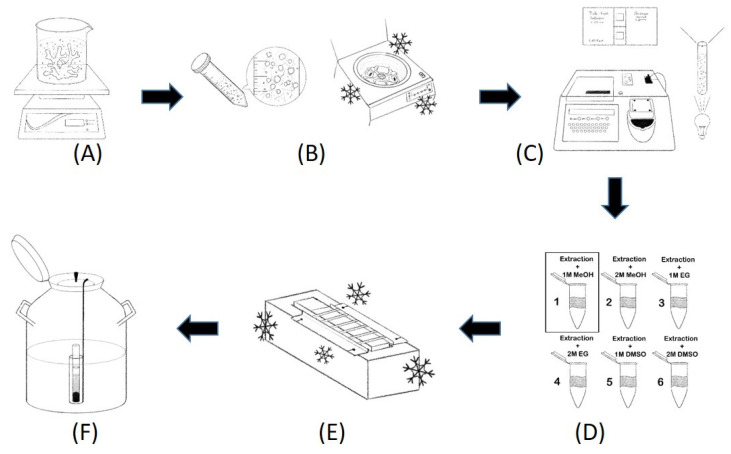
Cryobanking of coral cells with optimal CPAs and equilibration times. (**A**) Coral cells were extracted. (**B**) Coral cells were centrifuged for collection. (**C**) Viability was tested using ATP assay and cell counting. (**D**) Extracted cells were mixed with optimal CPA for optimal equilibration time. (**E**) Cells in straws were cooled through two-step cryopreservation. (**F**) Straws were immersed in liquid nitrogen for long-term preservation.

Coral cells were cryobanked in 0.5-mL straws (IMV Technologies, Normandy, France) using the optimal freezing conditions determined through the aforementioned assessment. Each straw was denoted by a four-digit number. The first digit indicated whether the coral was cultured or wild, the second and third digits, respectively, indicated the genus and species of the coral, and the fourth digit indicated the type of coral (e.g., massive, branching, foliaceous, encrusting, columnar, laminar, or free-living). The straws were inserted into a goblet mounted on an aluminum cane in a numbered cannister, which was later inserted into the stainless-steel canister of a cryogenic storage system (GT38 Air Liquide, Cryopal, France) for long-term storage (Figure 2).

### 2.7. Statistical Analysis

Statistical analysis was performed in SPSS (version 17.0; SPSS, Illinois, USA). The one-sample Kolmogorov–Smirnov test and Levene test were used to verify the normality and homogeneity of the data. A one-way analysis of variance and Least Significant Difference’s post hoc test were then performed to identify differences associated with the type of CPA, CPA concentration, and equilibration time. The data are presented as means ± standard errors of three replicates; a *p* of <0.05 was considered significant.

## 3. Results

### 3.1. Coral Cell Types

**Figure 3:**
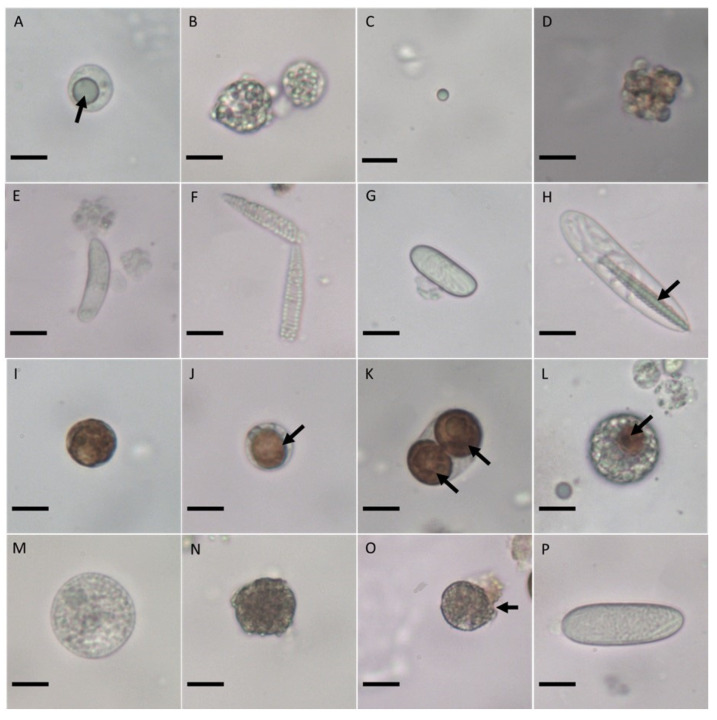
Extraction of multiple cell types through chemical dissolution before cryopreservation. Cells were generally colorless. Mucus cells formed in (**A**) single- and (**B**) multiple-vesicle gland cells, and host supporting cells were identified in (**C**) singular and (**D**) cluster form. Epidermal nematocysts were (**E**) crescent shaped and translucent, (**F**) elongated with a spiral tubule inner membrane (spirocyst), (**G**) capsule shaped with a tubular inner structure, or (**H**) needle shaped and encapsulated in a thread-like coil (arrow). (**I**) Symbiodiniaceae, which are round, brown cells, were present, and a host lipid body harbored (**J**) single and (**K**) multiple Symbiodiniaceae, known collectively as symbiotic endoderm cells (SECs). (**L**) Spindle cells with Symbiodiniaceae (arrow). (**M**) clustered cells in normal form. (**N**) Disintegrated Symbiodiniaceae, which were a dark, greenish color similar to that of the (**O**) ruptured cells (arrow). (**P**) Gastrodermal nematocysts with tubules wired inside the membrane and no shaft were also identified. Scale bar = 10 µm.

The coral tissue was composed of spherical gland cells with single (Figure 3A) or multiple vesicles (Figure 3B), which secrete mucus. Supporting cells are the key components of the epidermis; they are host cells found in both singular (Figure 3C) and cluster form (Figure 3D). All cnidaria species contain epidermal nematocysts, which enable predation; in this study, they were present as microbasic *p*-mastigophores in the shape of a crescent (Figure 3E), spiral (Figure 3F), capsule (Figure 3G), or encapsulated needle (Figure 3H). Symbiodiniaceae (Figure 3I) were frequently discovered in symbiotic coral. The SECs comprised single or multiple Symbiodiniaceae (Figure 3J,K) and were harbored in a layer of host lipid bodies, which enable photosynthesis. SECs are unique in that their host lipid bodies can expand to accommodate Symbiodiniaceae (Figure 3K). Spindle cells (cells that overlap) were found with the Symbiodiniaceae (Figure 3L) and had a similar shape and size to those of gland cells. Cluster cells were observed aligned in their normal form (Figure 3M). Disintegrated Symbiodiniaceae, which had dark, greenish coloration, were dispersed within the cells (Figure 3N). Ruptured cluster cells with disintegrated Symbiodiniaceae leaking into their inner cells were also identified (Figure 3O). Gastrodermal nematocysts (holotrichs) were only found in the gastrodermis; they appeared as tubules throughout and without a shaft (Figure 3P).

### 3.2. Cryopreservation of Coral Cells

Data on the coral species cryopreserved with different CPA types, CPA concentrations, equilibrium times, and extraction durations as well as the coral shapes and straw numbers are listed in Table 1. Each coral underwent the experiment individually, and an ATP assay and cell count were required for CPA suitability assessment. The results revealed DMSO was a suitable CPA for approximately half of the included corals (e.g., for the genera *Cyphastrea*, *Favia*, *Favites*, *Montipora*, *Pavona*, *Pocillopora*, *Porites*, *Echinophyllia*, *Lobophyllia*, *Turbinaria*, *Symphyllia*, *Seriatopora*, and *Merulina*), and successful cell cryopreservation for the other coral species was achieved using MeOH (e.g., for species within the genera *Acropora*, *Caulastrea*, *Echinopora*, *Hydnophora*, *Heliopora*, *Montipora*, *Merulina*, *Porites*, *Platygyra*, *Physogyra*, *Symphyllia*, and *Turbinaria*) and EG (e.g., for species within the genera *Acanthastrea*, *Acropora*, *Anacropora*, *Echinopora*, *Echinophyllia*, *Euphyllia*, *Favites*, *Heliopora*, *Hydnophora*, *Montastrea*, *Porites*, *Pavona*, *Sinularia*, *Seriatopora*, and *Turbinaria*). EG and DMSO were similarly suitable for preserving *Acanthastrea*, *Euphyllia*, *Favites*, *Lobophyllia*, *Pavona*, *Seriatopora*, and *Turbinaria*. EG and MeOH were similarly suitable for preserving *Acropora*, *Echinophyllia*, and *Sinularia*. Finally, MeOH and DMSO were similarly suitable for preserving *Platygyra*. Notably, all three CPAs (DMSO, EG, and MeOH) were suitable for approximately a quarter of the coral species (*A. tenuis*, *Anacropora forbesi*, *Cyphastrea ocellina*, *Caulastrea furcata*, *Echinopora lamellosa*, *Euphyllia paraancora*, *Heliopora coerulea*, *Montipora verrucosa*, *Pavona clavus*, *Pavona cactus*, *Porites lobata*, *Porites lutea*, *Platygyra pini*, *Porites nigrescens*, *Symphyllia recta*, *Turbinaria reniformis*, *Turbinaria mesenterina*, *Turbinaria sp. 1*, *Turbinaria peltata*, *Turbinaria stellulata*, and all *Montastrea* species) with both equilibration times. Symbiodiniaceae accounted for less than 10% of the total cells. Corals such as *Cyphastrea serailia*, *C. ocellina*, *Favia stelligera*, *Favia pallida*, *Favites flexuosa*, *H. coerulea*, *Hydnophora exesa*, *Hydnophora microconos*, *Leptoseris foliosa*, *Lithophyllon undulatum*, *Lobophyllia hemprichii*, *Montastrea valenciennesi*, *P. clavus*, *Symphyllia radians*, and *T. stellulata* had moderate viability (>50%) when the CPAs were used for freezing. A few corals had high viability (>70%) after cryopreservation, including *F. stelligera* and *F. pallida* in 1 M DMSO; *C. serailia* and *S. radians* in 2 M DMSO; and *F. flexuosa* in 1 and 2 M EG, 1 M DMSO, and 2 M MeOH. However, some corals had low viability (<10%) after freezing, including *Acropora azurea*, *Acropora subulate*, *Favites abdita*, *M. millepora*, *Montipora grisea*, *Montipora informis*, *Platygyra daedalea*, *Turbinaria frondens*, and *Acanthastrea* and *Hydnophora* species.

Both 10 and 20 min of equilibration time resulted in a 5–20% change in coral cell viability after freezing. The extraction duration for the coral ranged from 30 to 76 min. Cells from corals such as *Anthelia glauca*, *Favia favus*, *Montipora altasepta*, *Montipora foliosa*, *Montipora aequituberculata*, *Pachyseris speciosa*, *Platygyra lamellina*, and *Pachyseris rugosa* were not successfully extracted because slime formation led to cell clumping. Cells from 100 coral species were cryobanked, and at least 14 straws were obtained from each species, for a total of 1884 straws (0.5 mL) with an average concentration of 6.4 × 10^6^/mL.

### 3.3. Identification of Cell Types

The cell types identified for each coral species are presented in Table 2. High ratios of gland cells, supporting cells, epidermal nematocysts, Symbiodiniaceae, and symbiotic endoderm cells (SECs) were discovered in more than 80 coral species, indicating that basic coral cells can be easily obtained in high concentrations through the aforementioned extraction process. Among the 101 coral species, approximately half comprised spindle and cluster cells. Gastrodermal nematocysts were the type of cell identified least often. This indicates that some coral cells in deeper layers can be extracted through our protocol.

Epidermal nematocysts were not extracted from *A*. *hemprichii*, *C*. *furcata*, *Echinophyllia aspera*, *Echinophyllia echinata*, *F*. *abdita*, *Fungia scruposa*, *Isopora palifera*, *Montipora mollis*, *Porites lichen*, *Seriatopora caliendrum*, *Stylophora pistillata*, *Sinularia flexibilis*, or *S*. *recta*. Among the corals, *Galaxea fascicularis* had the highest ratio epidermal nematocysts. Symbiodiniaceae were extracted from every coral except *Cirrhipathes* sp. 1, which is a deep-sea coral. SECs are host cells comprising Symbiodiniaceae. SECs must be present in every zooxanthellate for coral to perform photosynthesis. However, corals such as *A*. *echinata*, *A*. *forbesi*, *C*. *furcata*, *E*. *aspera*, *E*. *echinata*, *E*. *paraancora*, *Euphyllia glabrescens*, *F*. *abdita*, *Favites complanata*, I. *palifera*, *Lobophyllia corymbosa*, *M*. *verrucosa*, *Montipora millepora*, *Pavona decussata*, *P*. *lichen*, *P*. *daedalea*, *Sinularia sadensis*, and *T*. *frondens* did not contain SECs. Spindle and cluster cells were present in half of the included corals and were easier to identify than gland and supporting cells were because of their inner structures and larger size. Gastrodermal nematocysts were identified in *A*. *azurea*, *Acropora subulata*, *Euphyllia glabrescens*, *G*. *fascicularis*, *H*. *coerulea*, *Hydnophora rigida*, *Pavona venosa*, *P*. *clavus*, *Platygyra ryukyuensis*, *Pocillopora verrucosa*, *P*. *lobata*, *Porites murrayensis*, and *P*. *lutea*

### 3.4. Symbiodiniaceae Concentration in Cells of Coral Species

**Figure 4:**
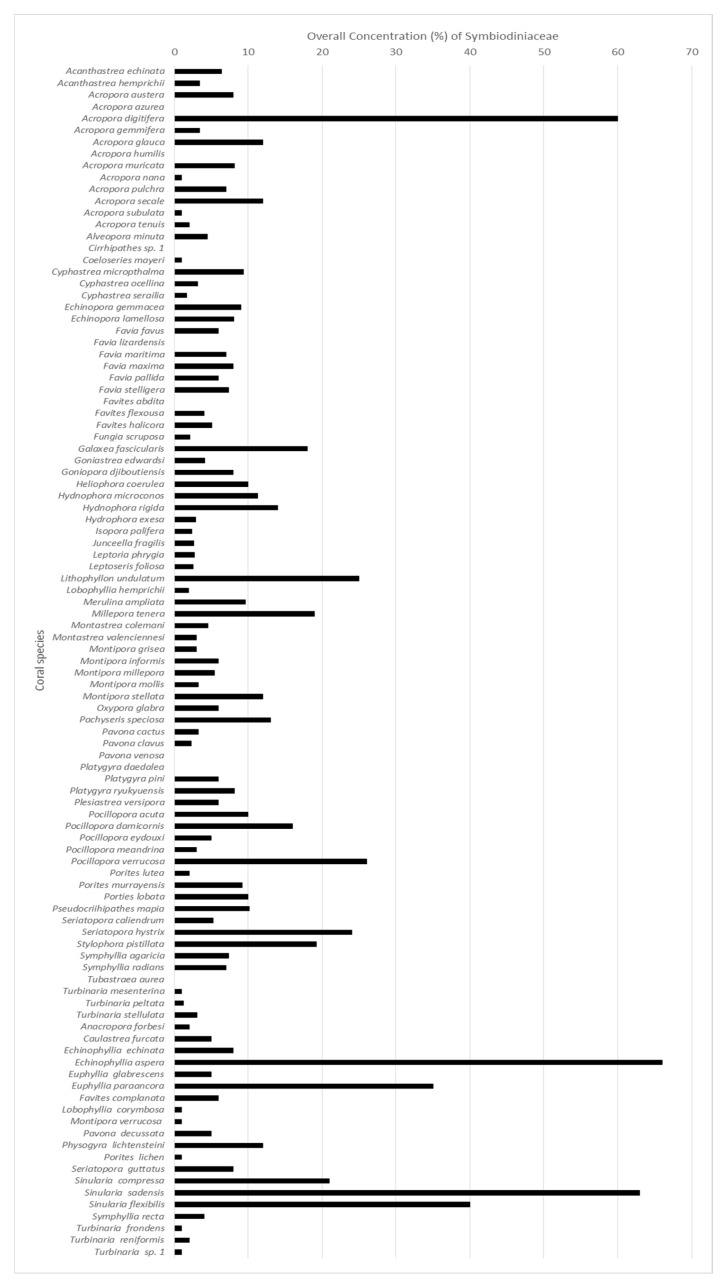
Overall Symbiodiniaceae concentration in cells of coral species after extraction.

The percentage of Symbiodiniaceae in each coral species is presented in Figure 4. More than 90% of the species contained Symbiodiniaceae. The remaining species, including the azooxanthellate coral *Tubastraea aurea*, had no Symbiodiniaceae. Notably, azooxanthellate coral do not contain or thus rely on Symbiodiniaceae to survive. Symbiodiniaceae were present in the coral cells at an average concentration of 8.6%. The five corals with the most abundant Symbiodiniaceae were *E. aspera*, *S. sadensis*, *S. flexibilis*, *E. paraancora*, and *P. verrucosa*. The lowest concentrations of Symbiodiniaceae were found in *A. subulata*, *Acropora nana*, *Coeloseris mayeri*, *L. corymbosa*, *M. verrucosa*, *T. mesenterina*, *T. peltata*, *Turbinaria* sp. *1*, and *T. frondens*. Different genus of corals comprised of various cell concentrations. These wild coral cells were extracted via chemical dissolution and counted using a hemocytometer, cell concentrations averaging at 7.3 × 10^6^ for each coral. For cultured coral, cell concentrations averaging at 5.4 × 10^6^ for each coral.

## 4. Discussion

Coral gametes, tissue balls, larvae, and Symbiodiniaceae were first cryopreserved through various techniques in the 2010s [51]. However, different coral materials may be more or less compatible with various freezing techniques. The size, shape, lipid content, and chilling sensitivity of the coral as well as the CPA and ice formation of the coral may affect the success of cryopreservation. Coral sperm have been extensively preserved because of the large volume of obtainable samples; such samples can be used for seeding purposes and contain abundant genetic biomaterial [52,53]. Coral sperm cryopreservation is commonly performed through two-step freezing with DMSO [10,19,54,55]. For coral oocytes, cryopreservation with MeOH has demonstrated promising results [56,57]. However, oocyte cryopreservation with MeOH requires vitrification with EG and propylene glycol [58,59] because high concentrations of MeOH can be toxic to oocytes. Nevertheless, attempts to cryopreserve asymbiotic [60] and symbiotic [61,62] coral larvae through vitrification and laser nanowarming have been successful.

CPAs enable cryopreservation at extremely low temperatures. The sperm of coral *Acropora humilis* have been successfully cryopreserved with 2 M DMSO [10], and oocytes from coral (e.g., *Echinopora* spp. and sp., *Junceella fragilis*, and *J. juncea*) might be suitably cryopreserved with 0.5 M MeOH [63], 1 M MeOH [64], and <3 M MeOH [55]. Tissue balls (*Pocillopora damicornis*) have been cryopreserved with ≤4 M EG, MeOH, glycogen (Gly), and DMSO and 1.5 M EG + 1.5 M Gly + 1.5 M DMSO [65,66]. Feuillassier et al. (2014) [65] also cryopreserved coral (*P. damicornis*) apices using 0.2 M sucrose + 0.75 M DMSO + 0.75 M MeOH + 0.75 M EG. The larvae of *F. scutaria* and *S. caliendrum* have been preserved with 10% PG + 5% DMSO + 1 M trehalose and 2 M EG + 1 M propylene glycol [60,61]. Finally, coral Symbiodiniaceae (e.g., *Symbiodinium*, *Breviolum*, *Cladocopium*, *Durusdinium*, *Fugacium*, and *Gerakladium* species) have been cryopreserved with 2 M EG, 2 M MeOH, 1 M Gly, 1 M MeOH, and 1 M MeOH + 0.4 M sucrose [11,14].

Membrane-permeating CPAs, such as DMSO, EG, and MeOH, have low molecular weights and can diffuse freely (if the equilibration time is sufficient) across membranes to protect cells from cold shock, chilling injury, and dehydration stress [64,67,68]. In this study, we discovered that no CPA could be applied to all coral species. However, DMSO, MeOH, and EG were suitable CPAs for several coral types. DMSO can strip water and metal ions [69], increase permeability by disintegrating bilayer structures [70], and prevent crystallization [71]. DMSO was effective on >50% of the corals in our experiments. DMSO is also suitable for coral sperm [10,19,72] and tissue balls [65,66]. By contrast, MeOH can prevent osmotic stress and preserve the gene expression, mitochondrial DNA, and lamina of nuclear envelopes [58,73]. In our study, MeOH was highly suitable for *H. microconos* (69%), *M. valenciennesi* (50%), and *Acropora muricata* (47%) after cryopreservation and was a suitable CPA for the genera *Acropora* and *Platygyra*. MeOH has also demonstrated effectiveness in the cryopreservation of the energy of coral (*Echinopora* sp.) oocytes [58] and the viability and fertility of coral (*J. juncea* and *J. fragilis*) sperm sacs [55] and was suitable for most Symbiodiniaceae, including *Symbiodinium*, *Breviolum*, *Cladocopium*, *Fugacium*, and *Gerakladium*, when used in a two-step vitrification and freezing method [14]. EG is similar to DMSO and MeOH with respect to membrane permeability and protection against fluorescence leakage [74,75,76]. In this study, EG was suitable for *P. clavus*, *H. microconos*, *Favites flexuosa*, and H. *coerulea* and achieved >50% viability after cryopreservation, indicating EG performed equally to MeOH. However, EG was less effective in protecting P. *damicornis* tissue balls against CPA toxicity than MeOH, glycerol, and DMSO were [65]. EG combined with other CPAs has been used in vitrification solutions for J. *juncea* oocytes (Tsai et al., 2015) and symbiotic coral (*S. caliendrum* and *P. verrucosa*) larvae cryopreserved through vitrification and laser nanowarming [33,61].

In this study, we identified morphological characteristics of coral cell types through chemical dissolution. Coral cells are found in two primary tissue layers, the external epidermis and internal gastrodermis [48,77,78]. Various cell types are restricted to a specific tissue layer [49]. Extraction through mechanical, chemical, or spontaneous means yields only basic cells, such as round cells (3–10 µm), Symbiodiniaceae (6–12 µm), SECs (10–15 µm), and nematocysts (15–20µm); this is supported by our results and those of several studies (Table 2; [35,38,47]). The cell density of extracted samples has been reported to range from 5 × 10^4^/mL to 5 × 10^7^/mL (Table 1; [24,43,47]). The average cell density was 6.4 × 10^6^/mL in this study.

Many aspects of coral cell function have been studied. Coral gland cells, which are secretory cells known as mucocytes [79,80], have been reported to transform into mucous cells [81] that function as antibacterial protectors, particle traps, and energy carriers [79,82,83]. In stony corals, supporting cells secrete a calcareous skeleton [84]. Only cnidaria have epidermal and gastrodermal nematocysts [85,86,87]. Morphological observations in the present study revealed that nematocysts have crescent-shaped, spirocyst, capsule-shaped, and encapsulated needle forms representing the developmental stages of coral tentacles, a finding supported by Ostman et al. (2010) [50]. Nematocysts play an essential role in defense against predators, locomotion, and host invasion [85,88,89]. SECs, which are Symbiodiniaceae combined with endodermal cells, enable nutrients and energy to flow between corals and hosts [90]. SECs have a unique capacity for expansion to enable the housing of more Symbiodiniaceae when necessary [49,91].

In the present study, 18 of 26 types of coral cell (e.g., granular gland cells, pigment cells, supporting cells, bipolar neurons, calicoblasts, desmocytes, epitheliomuscular cells, neurons, interstitial cells, interstitial stem cells, absorptive cells, and nutritive–muscular cells) were not identified. These coral cell types can be identified through single-cell RNA sequencing, scanning electron microscopy, transmission electron microscopy, histology, and the use of cell markers [37,45,46,48,84,92,93,94,95]. Rosental et al. (2017) [95] and Synder et al. (2020) [96] have used fluorescence-activated cell sorting to separate symbiotic and asymbiotic populations in in vitro cultures. However, this method is limited to identifying these two main populations. Single-cell RNA sequencing is the most effective method for studying cell–cell interactions and cell morphology and physiology in nonmodel species [48] and for identifying cell markers to screen for compounds indicative of coral cell functions [95].

In our study, small and round gland and supporting cells were abundant after cryopreservation. This can be attributed to their size (3–10 µm) and shape. Host coral cells are generally smaller (3–15 µm) and have a low permeability rate (1–10 µm^2^/s). Teardrop-shaped cells protrude and hinder diffusion because of their polarity; this does not occur in circular cells [97,98].

Some (<10%) of the Symbiodiniaceae extracted from our sampled corals were not the target cells; however, this did not affect the cellular ATP results. The 20-min equilibration time and 10-min holding time were insufficient for Symbiodiniaceae cryopreservation through the two-step freezing process. The appropriate equilibration times for the Symbiodiniaceae cryopreserved using two-step freezing with a 50 °C/min–100 °C/min cooling rate differ for different clades, such as *Symbiodinium* (30–60 min), *Breviolum* (20 min), *Cladocopium* (30 min), *Durusdinium* (30 min), *Fugacium* (30–60 min), and *Gerakladium* (30 min; Di Genio et al., 2021). Slightly longer equilibration times result from diffusion constraints caused by Symbiodiniaceae walls, both in hospite and in culture [99,100].

Various viability tests have been conducted on coral biomaterials using fluorescent metabolism markers [39], formazan spectrophotometry [43,101,102], flow cytometry with SYTOX Green [103], ATP assay [104], trypan blue exclusion testing [105], Neubauer chambers [106], fluorescein diacetate, and conventional propidium iodide [56,63]. In the present study, we used an ATP bioassay because of its ability to accurately and rapidly assess viability in only a few coral samples [11,14,33,61,71,103]. The assay detects ATP from the light emitted from the reaction of luciferin and luciferase [107,108]. Cell density was also calculated and may be an ideal proxy for determining coral health.

Mucus secretion, coral collection, and seasonal variation may have limited our experiment. Excessive mucus secretion can cause coral cells to coagulate, resulting in energy loss [79] and enhanced bacterial growth [109]. In this study, *Favia speciosa*, F. *favus*, M. *foliosa*, M. *altasepta*, P. *speciosa*, and *Platygyra lamellina* demonstrated signs of excessive mucus buildup that trapped cells, rendering impossible the separation of cells for cryopreservation. Furthermore, the collection site experiences drastic temperature variations due to a nearby power plant, strong tidally induced upwellings, and typhoons [110]. High temperatures can affect coral Symbiodiniaceae by increasing reactive oxygen species production, which can lead to oxidative stress [111,112]. An increase in sea surface temperature occurred from May to August and led to mass coral bleaching at Houbihu, Kenting. Most of the corals at the site were bleached, thus preventing coral collection from August to November, until the area demonstrated signs of recovery. Although the corals recovered, their ability to withstand cryopreservation may have been weakened because their energy was focused on recuperation after bleaching [113]. Increases in sea surface temperatures and the frequency of bleaching events may challenge the survival of coral species.

This was the first study on coral cell cryopreservation and cryobanking in which various CPAs, CPA concentrations and equilibration times were applied to numerous coral species. The cells of 100 coral species were cryobanked, with at least 14 straws for each species, for a total of 1884 straws (0.5 mL) with minimum concentrations of 1 × 10^6^/mL. To sustain the world’s coral reefs, cryobanking coral cells is crucial; cryobanked cells can be used for reculture, nuclear transfer cloning, recombinant DNA, gene delivery, and genome editing. Cryobanking is a new form of coral cell preservation; the results of this study may serve as a framework for cryobanks worldwide and may contribute to the long-term conservation of coral reefs.

## Figures and Tables

**Table 1:** Cryopreserved wild (a) and cultured (b) coral cell data, including coral shape, suitable CPA, viability rate (%), equilibrium time (min), extraction duration (min), and number of straws.

(a)									
No	Genus	Species	Shape	Suitable CPA	Viability Rate (%)	Equilibrium Time (min)	Extraction Duration (min)	No of Straws	ANOVA
1	*Acanthastrea*	*echinata*	Massive	1M EG	6 ± 0.8	20	30	31	F_12,26_ = 2.259, *p* < 0.05
2	*Acanthastrea*	*hemprichii*	Massive	1M DMSO	9 ± 1.7	10	30	16	F_12,26_ = 29.722, *p* < 0.001
3	*Acropora*	*austera*	Branching	1 M MeOH	15 ± 1.3	10	30	16	F_12,26_ = 50.236, *p* < 0.001
4	*Acropora*	*azurea*	Branching	1M EG	8 ± 0.5	10	30	15	F_12,26_ = 102.923, *p* < 0.001
5	*Acropora*	*digitifera*	Branching	2 M DMSO	16 ± 1.0	10	30	15	F_12,26_ = 9.342, *p* < 0.001
6	*Acropora*	*gemnifera*	Branching	1M EG	25 ± 7.7	10	35	15	F_12,26_ = 60.145, *p* < 0.001
7	*Acropora*	*glauca*	Branching	1M DMSO	29 ± 0.0	20	30	16	F_12,26_ = 43.432, *p* < 0.001
8	*Acropora*	*humilis*	Branching	2M DMSO	18 ± 4.8	10	30	15	F_12,26_ = 84.850, *p* < 0.001
9	*Acropora*	*muricata*	Branching	2M MeOH	41 ± 12.6	20	30	47	F_12,26_ = 5.144, *p* < 0.001
10	*Acropora*	*nana*	Branching	2M MeOH	38 ± 8.3	20	30	47	F_12,26_ = 79.298, *p* < 0.001
11	*Acropora*	*pulchra*	Branching	1M MeOH	22 ± 0.3	20	30	16	F_12,26_ = 239.681, *p* < 0.001
12	*Acropora*	*secale*	Branching	1M EG	33 ± 0.1	20	30	16	F_12,26_ = 25.654, *p* < 0.001
13	*Acropora*	*subulata*	Branching	1M EG	7 ± 0.4	20	30	16	F_12,26_ = 749.775, *p* < 0.001
14	*Acropora*	*tenuis*	Branching	1M DMSO	49 ± 1.2	10	30	16	F_12,26_ = 70.120, *p* < 0.001
15	*Alveopora*	*minuta*	Massive	2M MeOH	25 ± 9.5	20	30	15	F_12,26_ = 6.152, *p* < 0.001
16	*Cirrhipathes*	*sp 1*	Spines	1M DMSO	13 ± 2.4	20	30	0	F_12,26_ = 554.545, *p* < 0.001
17	*Coeloseries*	*mayeri*	Massive	2M MeOH	7 ± 1.5	20	30	15	F_12,26_ = 5.749, *p* < 0.001
18	*Cyphastrea*	*micropthalma*	Encrusting	1M DMSO	28 ± 9.6	20	30	16	F_12,26_ = 2.377, *p* < 0.05
19	*Cyphastrea*	*Ocellina*	Encrusting	2M EG or 1M DMSO	56 ± 12.0	20	30	16	F_12,26_ = 21.547, *p* < 0.001
20	*Cyphastrea*	*serailia*	Massive	2M DMSO	75 ± 15.9	20	30	16	F_12,26_ = 3.037, *p* < 0.05
21	*Echinopora*	*gemmacea*	Encrusting	1M MeOH	8 ± 1.5	10	60	16	F_12,26_ = 103.324, *p* < 0.001
22	*Echinopora*	*lamellosa*	Foliaceous	2M EG	13 ± 4.7	20	60	16	F_12,26_ = 62.706, *p* < 0.001
23	*Favia*	*favus*	Massive	1M DMSO	16 ± 1.8	10	30	15	F_12,26_ = 25.545, *p* < 0.001
24	*Favia*	*lizardensis*	Massive	1M DMSO	27 ± 13.6	20	30	31	F_12,26_ = 8.990, *p* < 0.001
25	*Favia*	*maritima*	Massive	1M DMSO	63 ± 15.4	10	30	15	F_12,26_ = 21.565, *p* < 0.001
26	*Favia*	*maxima*	Massive	1M DMSO	60 ± 24.7	20	30	16	F_12,26_ = 76.453, *p* < 0.001
27	*Favia*	*pallida*	Massive	1M DMSO	53 ± 21.5	20	30	15	F_12,26_ = 2.511, *p* < 0.05
28	*Favia*	*stelligera*	Massive	2M DMSO	96 ± 15.0	10	30	15	F_12,26_ = 111.196, *p* < 0.001
29	*Favites*	*abdita*	Massive	1M DMSO	6 ± 0.5	10	30	30	F_12,26_ = 3209.843, *p* < 0.001
30	*Favites*	*flexousa*	Massive	1M EG	73 ± 32.5	20	30	16	F_12,26_ = 27.451, *p* < 0.001
31	*Favites*	*halicora*	Massive	1M DMSO	34 ± 8.6	10	30	16	F_12,26_ = 21.884, *p* < 0.001
32	*Fungia*	*scruposa*	Massive	1M MeOH	10 ± 1.0	20	30	30	F_12,26_ = 458.454, *p* < 0.001
33	*Galaxea*	*fascicularis*	Massive	2M DMSO	10 ± 5.7	10	30	14	F_12,26_ = 13.064, *p* < 0.001
34	*Goniastrea*	*edwardsi*	Massive	1M DMSO	9 ± 0.2	10	30	47	F_12,26_ = 4818.927, *p* < 0.001
35	*Goniopora*	*djiboutiensis*	Massive	1M MeOH	39 ± 0.1	10	30	16	F_12,26_ = 6.654, *p* < 0.001
36	*Heliopora*	*coerulea*	Massive	1M EG	49 ± 5.0	10	76	16	F_12,26_ = 49.548, *p* < 0.001
37	*Hydnophora*	*exesa*	Massive	1M DMSO	50 ± 10.5	20	30	16	F_12,26_ = 47.386, *p* < 0.001
38	*Hydnophora*	*microconos*	Branching	2M MeOH	19 ± 5.8	10	30	15	F_12,26_ = 2.295, *p* < 0.05
39	*Hydnophora*	*rigida*	Encrusting	1M EG	12 ± 1.6	10	30	15	F_12,26_ = 203.269, *p* < 0.001
40	*Isopora*	*palifera*	Laminar	2M DMSO	18 ± 6.7	10	35	16	F_12,26_ = 53.385, *p* < 0.001
41	*Junceella*	*fragilis*	Columnar	1M EG	12 ± 1.8	20	30	15	F_12,26_ = 517.381, *p* < 0.001
42	*Leptoria*	*phrygia*	Massive	1M DMSO	35 ± 8.3	20	30	16	F_12,26_ = 8.106, *p* < 0.001
43	*Leptoseries*	*foliosa*	Encrusting	1M DMSO	50 ± 3.5	20	30	15	F_12,26_ = 22.000, *p* < 0.001
44	*Lithophyllon*	*undulatum*	Encrusting	1M DMSO	66 ± 17.3	20	35	16	F_12,26_ = 54.412, *p* < 0.001
45	*Lobophyllia*	*hemprichii*	Massive	2M DMSO	55 ± 17.5	20	30	16	F_12,26_ = 10.507, *p* < 0.001
46	*Merulina*	*ampliata*	Foliaceous	1M DMSO	40 ± 11.6	10	30	15	F_12,26_ = 140.370, *p* < 0.001
47	*Millepora*	*tenera*	Branching	1M MeOH	21 ± 1.8	20	35	30	F_12,26_ = 28.980, *p* < 0.001
48	*Montastrea*	*colemani*	Encrusting	1M EG	41 ± 7.0	10	30	30	F_12,26_ = 49.303, *p* < 0.001
49	*Montastrea*	*valenciennesi*	Massive	1M MeOH	57 ± 16.8	10	30	16	F_12,26_ = 4.168, *p* = 0.001
50	*Montipora*	*grisea*	Foliaceous	1M DMSO	8 ± 2.8	10	30	16	F_12,26_ = 26.440, *p* < 0.001
51	*Montipora*	*informis*	Encrusting	1M DMSO	2 ± 0.4	10	30	15	F_12,26_ = 48.325, *p* < 0.001
52	*Montipora*	*millepora*	Foliaceous	1M MeOH	10 ± 4.7	20	30	15	F_12,26_ = 18.521, *p* < 0.001
53	*Montipora*	*mollis*	Foliaceous	1M DMSO	25 ± 3.2	10	30	16	F_12,26_ = 66.964, *p* < 0.001
54	*Montipora*	*stellata*	Branching	1M DMSO	12 ± 2.3	10	30	15	F_12,26_ = 15.343, *p* < 0.001
55	*Oxypora*	*glabra*	Encrusting	1M DMSO	28 ± 4.3	10	30	15	F_12,26_ = 53.656, *p* < 0.001
56	*Pachyseris*	*speciosa*	Encrusting	1M DMSO	23 ± 6.2	10	30	15	F_12,26_ = 15.573, *p* < 0.001
57	*Pavona*	*cactus*	Massive	1M DMSO	16 ± 7.7	20	40	46	F_12,26_ = 127.857, *p* < 0.001
58	*Pavona*	*clavus*	Branching	2M EG or 1M DMSO	53 ± 8.9	20 and 10	30	15	F_12,26_ = 18.187, *p* < 0.001
59	*Pavona*	*venosa*	Columnar	1M DMSO	17 ± 18.5	10	30	31	F_12,26_ = 39.809, *p* < 0.001
60	*Platygyra*	*daedalea*	Massive	2M DMSO	9 ± 1.1	10	45	15	F_12,26_ = 9.891, *p* < 0.001
61	*Platygyra*	*pini*	Massive	1M MeOH	82 ± 11.5	10	30	15	F_12,26_ = 39.283, *p* < 0.001
62	*Platygyra*	*ryukyuensis*	Massive	1M MeOH	29 ± 9.3	10	30	45	F_12,26_ = 1.866, *p* < 0.001
63	*Plesiastrea*	*versipora*	Massive	1M DMSO	11 ± 1.7	10	30	30	F_12,26_ = 396.433, *p* < 0.001
64	*Pocillopora*	*acuta*	Branching	1M DMSO	9 ± 0.5	10	30	15	F_12,26_ = 6.448, *p* < 0.001
65	*Pocillopora*	*damicornis*	Branching	1M DMSO	21 ± 2.9	10	40	31	F_12,26_ = 410.839, *p* < 0.001
66	*Pocillopora*	*eydouxi*	Branching	1M DMSO	3 ± 1.1	20	50	16	F_12,26_ = 18.488, *p* < 0.001
67	*Pocillopora*	*meandrina*	Branching	1M DMSO	6 ± 1.1	10	50	16	F_12,26_ = 65.020, *p* < 0.001
68	*Pocillopora*	*verrucosa*	Branching	1M DMSO	16 ± 4.3	10	30	16	F_12,26_ = 49.115, *p* < 0.001
69	*Porites*	*lobata*	Massive	2M DMSO	17 ± 1.4	10	30	16	F_12,26_ = 373.436, *p* < 0.001
70	*Porites*	*lutea*	Massive	1M MeOH	25 ± 4.1	20	30	16	F_12,26_ = 84.609, *p* < 0.001
71	*Porites*	*murrayensis*	Massive	1M DMSO	10 ± 3.2	20	30	16	F_12,26_ = 20.322, *p* < 0.001
72	*Pseudocriihipathes*	*mapia*	Columnar	2M DMSO	23 ± 4.1	10	40	16	F_12,26_ = 157.210, *p* < 0.001
73	*Seriatopora*	*caliendrum*	Branching	1M DMSO	23 ± 7.0	20	30	16	F_12,26_ = 66.962, *p* < 0.001
74	*Seriatopora*	*hystrix*	Branching	1 M MeOH	63 ± 0.1	10	30	16	F_12,26_ = 8.843, *p* < 0.001
75	*Stylophora*	*pistillata*	Branching	1M EG	17 ± 5.5	10	75	16	F_12,26_ = 58.211, *p* < 0.001
76	*Symphyllia*	*agaricia*	Massive	2M MeOH	8 ± 1.5	10	35	16	F_12,26_ = 71.498, *p* < 0.001
77	*Symphyllia*	*radians*	Massive	2M DMSO	71 ± 15.3	10	30	30	F_12,26_ = 2.322, *p* < 0.05
78	*Tubastraea*	*aurea*	Massive	1M DMSO	12 ± 1.5	10	30	16	F_12,26_ = 126.703, *p* < 0.001
79	*Turbinaria*	*mesenterina*	Foliaceous	1M EG	12 ± 2.6	10	30	15	F_12,26_ = 58.834, *p* < 0.001
80	*Turbinaria*	*peltata*	Foliaceous	2M EG	29 ± 4.8	10	30	32	F_12,26_ = 194.098, *p* < 0.001
81	*Turbinaria*	*stellulata*	Foliaceous	1M DMSO	77 ± 43	20	30	15	F_12,26_ = 4.270, *p* = 0.001
(**b**)									
**No**	Genus	Species	Shape	Suitable CPA	Viability rate (%)	Equilibrium time (min)	Extraction duration (min)	No of straws	ANOVA
1	*Anacropora*	*forbesi*	Columnar	2 M EG	39 ± 8.5	10	30	15	F_12,26_ = 2.905, *p* < 0.05
2	*Caulastrea*	*furcata*	Massive	1M MeOH	22 ± 1.3	10	30	47	F_12,26_ = 832.591, *p* < 0.001
3	*Echinophyllia*	*aspera*	Laminar	1M EG	41 ± 12.3	10	30	30	F_12,26_ = 28.475, *p* < 0.001
4	*Echinophyllia*	*echinata*	Massive	1M DMSO	39 ± 5.6	10	30	31	F_12,26_ = 211.678, *p* < 0.001
5	*Euphyllia*	*glabrescens*	Branching	1 M EG	3 ± 0.6	10	30	15	F_12,26_ = 2405.531, *p* < 0.001
6	*Euphyllia*	*paraancora*	Branching	1M EG	23 ± 0.7	10	30	29	F_12,26_ = 244.752, *p* < 0.001
7	*Favites*	*complanata*	Columnar	1M DMSO	12 ± 1.6	10	30	32	F_12,26_ = 623.795, *p* < 0.001
8	*Lobophyllia*	*corymbosa*	Massive	1M DMSO	28 ± 7.8	10	30	32	F_12,26_ = 45.394, *p* < 0.001
9	*Montipora*	*verrucosa*	Foliaceous	1M MeOH	20 ± 5.0	20	30	16	F_12,26_ = 73.686, *p* < 0.001
10	*Pavona*	*decussata*	Foliaceous	1M DMSO	36 ± 12	10	30	15	F_12,26_ = 103.974, *p* < 0.001
11	*Physogyra*	*lichtensteini*	Massive	1M MeOH	19 ± 6.4	10	30	15	F_12,26_ = 179.699, *p* < 0.001
12	*Porites*	*lichen*	Foliaceous	2M EG	21 ± 1.6	10	30	32	F_12,26_ = 165.086, *p* < 0.001
13	*Seriatopora*	*guttatus*	Branching	1M EG	17 ± 3.5	10	30	16	F_12,26_ = 298.055, *p* < 0.001
14	*Sinularia*	*compressa*	Branching	1M EG	17 ± 2.4	10	30	16	F_12,26_ = 481.321, *p* < 0.001
15	*Sinularia*	*flexibilis*	Branching	1M EG	13 ± 1.6	10	30	30	F_12,26_ = 1652.858, *p* < 0.001
16	*Sinularia*	*sadensis*	Branching	2M EG	24 ± 3.5	10	30	15	F_12,26_ = 434.807, *p* < 0.001
17	*Symphyllia*	*recta*	Massive	1M DMSO	18 ± 4.4	20	30	16	F_12,26_ = 19.750, *p* < 0.001
18	*Turbinaria*	*frondens*	Branching	1M EG	8 ± 2.5	20	30	32	F_12,26_ = 82.402, *p* < 0.001
19	*Turbinaria*	*reniformis*	Foliaceous	2M MeOH	25 ± 3.4	20	30	16	F_12,26_ = 41.255, *p* < 0.001
20	*Turbinaria*	*sp 1*	Foliaceous	1M MeOH	27 ± 2.9	10	35	31	F_12,26_ = 244.116, *p* < 0.001

-*Cirrhipathes sp. 1* was not cryobanked because the deep-sea coral samples were insufficient. –No 1–20 were corals cultured for more than 3 years at the NMMBA.

**Table 2:** Identification of wild (a) and cultured (b) cell types within two different tissue layers in 101 coral species.

(a)										
			Cell Types Identification							
No	Genus	Species	Gland Cell	Supporting Cell	Epidermal Nematocyst	Symbiodiniaceae	Symbiotic Endoderm Cell (SEC)	Spindle Cell	Cluster Cell	Gastrodermal Nematocyst
1	*Acanthastrea*	*echinata*	●	●	●	●			●	
2	*Acanthastrea*	*hemprichii*	●	●		●	●	●	●	
3	*Acropora*	*austera*	●	●	●	●			●	
4	*Acropora*	*azurea*	●	●	●	●	●	●	●	●
5	*Acropora*	*digitifera*		●		●				●
6	*Acropora*	*gemnifera*	●	●	●	●	●		●	
7	*Acropora*	*glauca*	●	●	●	●	●			●
8	*Acropora*	*humilis*	●	●	●	●	●	●	●	
9	*Acropora*	*muricata*	●	●	●	●	●			
10	*Acropora*	*nana*	●	●	●	●	●			
11	*Acropora*	*pulchra*	●	●	●	●		●		
12	*Acropora*	*secale*	●	●	●	●				●
13	*Acropora*	*subulata*	●	●	●	●	●			●
14	*Acropora*	*tenuis*	●	●	●	●	●	●	●	●
15	*Alveopora*	*minuta*	●	●	●	●	●	●	●	
16	*Cirrhipathes*	*sp 1*	●	●	●		●	●	●	
17	*Coeloseries*	*mayeri*	●	●	●	●	●			
18	*Cyphastrea*	*micropthalma*	●	●	●	●	●			
19	*Cyphastrea*	*Ocellina*	●	●	●	●	●			
20	*Cyphastrea*	*serailia*	●	●	●	●	●		●	
21	*Echinopora*	*gemmacea*	●	●	●	●	●	●	●	
22	*Echinopora*	*lamellosa*	●	●	●	●	●			
23	*Favia*	*favus*	●	●	●	●	●		●	
24	*Favia*	*lizardensis*	●	●	●	●	●	●	●	
25	*Favia*	*maritima*	●	●	●	●	●	●	●	●
26	*Favia*	*maxima*	●	●	●	●	●			
27	*Favia*	*pallida*	●	●	●					●
28	*Favia*	*stelligera*	●	●	●	●	●		●	
29	*Favites*	*abdita*	●	●		●			●	
30	*Favites*	*flexousa*	●	●	●	●	●		●	
31	*Favites*	*halicora*	●	●	●	●	●	●	●	
32	*Fungia*	*seruposa*	●	●		●	●		●	
33	*Galaxea*	*fascicularis*	●	●	●	●	●	●		●
34	*Goniopora*	*djiboutiensis*	●	●	●	●	●			●
35	*Goniastrea*	*edwardsi*	●	●	●	●	●	●	●	
36	*Heliopora*	*coerulea*	●	●	●	●	●	●	●	●
37	*Hydnophora*	*exesa*	●	●	●	●	●	●		
38	*Hydnophora*	*microconos*	●	●	●	●	●	●		
39	*Hydnophora*	*rigida*	●	●	●	●	●	●		●
40	*Isopora*	*palifera*	●	●		●				
41	*Junceella*	*fragilis*	●	●	●	●	●			
42	*Leptoria*	*phrygia*	●	●	●	●	●	●		
43	*Leptoseries*	*foliosa*	●	●	●	●	●			
44	*Lithophyllon*	*undulatum*	●	●	●	●	●	●		
45	*Lobophyllia*	*hemprichii*	●	●	●	●	●		●	
46	*Merulina*	*ampliata*	●	●	●	●	●		●	
47	*Millepora*	*tenera*	●	●	●	●	●		●	
48	*Montastrea*	*colemani*	●	●	●	●	●			
49	*Montipora*	*grisea*	●	●	●	●	●			
50	*Montipora*	*informis*	●	●	●	●				
51	*Montipora*	*millepora*	●	●	●	●			●	
52	*Montipora*	*mollis*	●	●		●	●			
53	*Montipora*	*stellata*	●	●		●	●	●	●	●
54	*Montastrea*	*valenciennesi*	●	●	●	●	●	●		
55	*Oxypora*	*glabra*	●	●	●	●	●	●	●	
56	*Pachyseris*	*speciosa*	●	●	●	●	●	●		●
57	*Pavona*	*cactus*	●	●	●	●	●	●		
58	*Pavona*	*clavus*	●	●	●	●	●	●	●	●
59	*Pavona*	*venosa*	●	●	●	●	●			●
60	*Platygyra*	*daedalea*	●	●	●	●		●	●	
61	*Platygyra*	*pini*	●	●	●	●				
62	*Platygyra*	*ryukyuensis*	●	●	●	●	●	●	●	●
63	*Plesiastrea*	*versipora*	●	●	●	●	●	●	●	
64	*Pocillopora*	*acuta*	●	●	●	●	●	●	●	●
65	*Pocillopora*	*damicornis*	●	●	●	●	●		●	
66	*Pocillopora*	*eydouxi*	●	●	●	●	●	●	●	
67	*Pocillopora*	*meandrina*	●	●	●	●	●	●	●	
68	*Pocillopora*	*verrucosa*	●	●	●	●	●	●		●
69	*Porites*	*lobata*	●	●	●	●	●		●	●
70	*Porites*	*lutea*	●	●	●	●	●		●	●
71	*Porites*	*murrayensis*	●	●	●	●	●			●
72	*Pseudocriihipathes*	*mapia*	●	●	●	●	●		●	●
73	*Seriatopora*	*caliendrum*	●	●		●	●		●	
74	*Seriatopora*	*hystrix*	●	●	●	●	●			●
75	*Stylophora*	*pistillata*	●	●		●	●			
76	*Symphyllia*	*agaricia*	●	●	●	●	●	●		
77	*Symphyllia*	*radians*	●	●	●	●				
78	*Tubastraea*	*aurea*	●	●	●	●	●	●	●	●
79	*Turbinaria*	*mesenterina*	●	●	●	●	●			●
80	*Turbinaria*	*peltata*	●	●	●	●	●			●
81	*Turbinaria*	*stellulata*	●	●	●	●	●	●		●
(**b**)										
			**Cell Types Identification**							
**No**	**Genus**	**Species**	**Gland Cell**	**Supporting Cell**	**Epidermal Nematocyst**	**Symbiodiniaceae**	**Symbiotic Endoderm Cell (SEC)**	**Spindle Cell**	**Cluster Cell**	**Gastrodermal Nematocyst**
1	*Anacropora*	*forbesi*	●	●	●	●				
2	*Caulastrea*	*furcata*	●	●		●				
3	*Echinophyllia*	*aspera*	●	●		●				
4	*Echinophyllia*	*echinata*	●	●		●				
5	*Euphyllia*	*glabrescens*	●	●	●	●				●
6	*Euphyllia*	*paraancora*	●	●	●	●			●	
7	*Favites*	*complanata*	●	●	●	●				
8	*Lobophyllia*	*corymbosa*	●	●	●	●				
9	*Montipora*	*verrucosa*	●	●		●			●	
10	*Pavona*	*decussata*	●	●	●	●				
11	*Physogyra*	*lichtensteini*	●	●	●	●	●		●	
12	*Porites*	*lichen*	●	●		●				
13	*Seriatopora*	*guttatus*	●	●	●	●	●			
14	*Sinularia	*compressa*								
15	*Sinularia*	*flexibilis*	●	●		●	●			
16	*Sinularia*	*sadensis*	●	●	●	●				
17	*Symphyllia*	*recta*	●			●	●			
18	*Turbinaria*	*frondens*	●	●	●	●				
19	*Turbinaria*	*reniformis*	●	●	●	●	●		●	
20	*Turbinaria*	*sp 1*	●	●	●	●	●		●	

● Availability of cells. Numbers 1–20 were corals cultured for more than 3 year at the NMMBA. * Data for *Sinularia compressa* unavailable.

## Data Availability

Not applicable.
